# Getting ready to cope with non-communicable eye diseases

**Published:** 2014

**Authors:** Clare Gilbert, Serge Resnikoff

**Affiliations:** Co-director: International Centre for Eye Health, Disability Group, London School of Hygiene and Tropical Medicine, London, UK.; President: International Health and Development, Geneva, Switzerland. Serge.resnikoff@gmail.com

**Figure:**
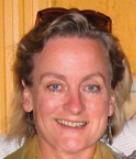
Clare Gilbert

**Figure:**
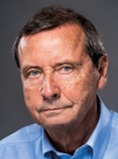
Serge Resnikoff

Over the last few decades, the main focus of eye care in low- and middle-income countries has been on the two most common causes of visual impairment: cataract and refractive errors. Although there are certainly challenges involved in providing high quality services at a scale that is able to meet the need, these two conditions are straightforward to manage. Screening is not required (apart from children with refractive errors), as both conditions are associated with a loss of vision. Patients are usually very happy with the outcome (surgery or spectacles) and minimal follow-up is needed.

Providing services for non-communicable eye diseases (NCEDs), however, is far more challenging and complex. To prevent visual loss from diabetic retinopathy, glaucoma and retinopathy of prematurity, for example, the disease must be detected before people themselves notice a problem. This requires screening or other approaches to case finding. Treatment to prevent sight loss needs to be given early, which means patients must be counselled (i.e., informed about their options and supported to make the decision that is right for them). Long-term follow-up is essential, for example for glaucoma, diabetic retinopathy (DR), and age-related macular degeneration (AMD).

## How can we respond?

In order to offer adequate services for NCEDs, the whole health system – as it applies to eye health – must be considered. The World Health Organization says the following about health systems: ‘A good health system delivers quality services to all people, when and where they need them.’

All organisations and agencies whose purpose is to improve health are part of the health system in a country; this includes government health services as well as those provided by not-for profit providers and the private, for-profit, sector – whether in the community or at primary, secondary/district or tertiary level.

The six building blocks of the health system are:

- leadership and governance
- the health workforce
- technology, equipment, infrastructure, and medicines
- health financing
- health management information systems
- service delivery.

In order to cope with NCEDs, the following is required at each of the six building blocks.

### Leadership and governance

- Evidence-based clinical protocols and guidelines.
- Good management of resources.
- Leadership and team-building (including forging links with other sectors or other health departments, e.g. endocrinology).

### Health workforce

- Staff at every level of service delivery who are competent in comprehensive eye examination, with appropriate treatment or referral to higher levels, thereby providing a continuum of care.
- Optometrists skilled in optic disc assessment and interpreting visual fields and detection of DR.
- Sufficient numbers of patient counsellors, supported by appropriate educational materials for patients.
- Technicians to protect, maintain and repair equipment (such as visual field analysers and lasers).
- Team work and task shifting to ensure good management, adequate visual field testing and screening using retinal imaging. For example, hospital optometrists can manage stable glaucoma patients and technicians can screen for DR using retinal imaging.
  **Figure:**
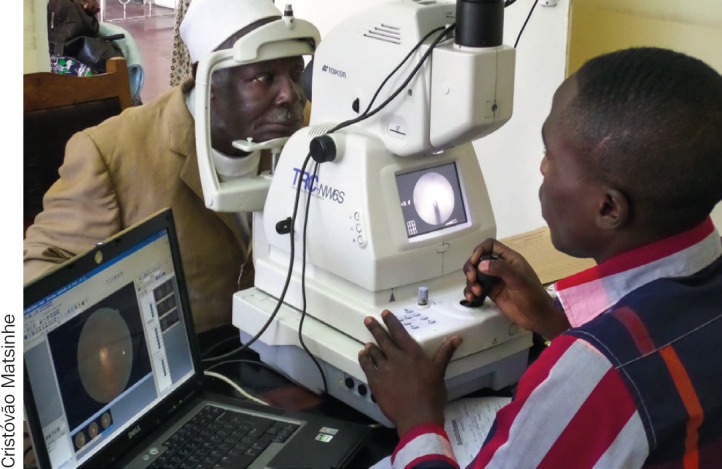
A photographer working with a mobile clinic team takes fundus images in a rural hospital. Pictures are graded by ophthalmologists and residents in the referral hospital. If need be, patients are informed and offered further examination and treatment in the referral hospital.
- Sub-specialty training in medical and surgical retina and glaucoma.

### Technology, equipment, infrastructure and medicines

- Fundus cameras, visual field analysers, lasers, indirect ophthalmoscopes and vitrectomy machines.
- Affordable medication for non-communicable eye diseases with well maintained stocks, including affordable medication for glaucoma.
- Telemedicine systems for remote interpretation of retinal images.

### Health financing

- Low cost medication.
- Insurance packages or other financing schemes for eye care which include treatment for glaucoma, diabetic retinopathy, age-related macular degeneration and retinopathy of prematurity.

### Health management information systems

- Electronic patient records.
- Good record keeping and retrieval systems.
- Monitoring rates of follow-up

### Service delivery

- Dedicated clinics for glaucoma and retina at tertiary level, where all team members are adequately trained.
- Good care pathways.
- Systems in place to encourage follow-up e.g. SMS messages.
- Relevant activities at primary, secondary and tertiary levels.
- Opportunistic screening for glaucoma in all outpatients aged 30 or 40 years and above.

### Conclusion

In order to overcome barriers, it is helpful to be responsive to the community and their needs. For example, hold clinics at times that suit working people (e.g. weekends and evenings) or screen for DR in physicians' clinics. Everyone providing eye care, at whatever level they work, can gain knowledge and skills to reduce visual loss from non-communicable eye diseases, which are an increasingly important cause of visual loss in all parts of the world.
